# NFκB1 Polymorphisms Are Associated with Severe Influenza A (H1N1) Virus Infection in a Canadian Population

**DOI:** 10.3390/microorganisms10101886

**Published:** 2022-09-21

**Authors:** Suhrobjon Mullo Mirzo, Anand Kumar, Naresh Kumar Sharma, Lin Li, Robert Balshaw, Francis A. Plummer, Ma Luo, Binhua Liang

**Affiliations:** 1 Max Rady College of Medicine, University of Manitoba, Winnipeg, MB R3X 0P3, Canada; 2 Department of Medical Microbiology and Infectious Diseases, University of Manitoba, Winnipeg, MB R3E 0J9, Canada; 3 Critical Care Medicine, Health Science Centre, University of Manitoba, Winnipeg, MB R3A 1R9, Canada; 4 JC Wilt Infectious Diseases Research Centre, National Microbiology Laboratory, Winnipeg, MB R3E 3L5, Canada; 5 Data Sciences Platform, George & Fay Yee Centre for Healthcare Innovation, University of Manitoba, Winnipeg, MB R3X 0T6, Canada; 6 Department of Biochemistry and Medical Genetics, University of Manitoba, Winnipeg, MB R3E 0J9, Canada

**Keywords:** NFκB1, H1N1, SNP, influenza, severe

## Abstract

Background: We examined associations between NFκB1 polymorphisms and influenza A (H1N1) clinical outcomes in Canadian. Methods: A total of thirty-six Caucasian patients admitted to the intensive care unit (ICU) in hospitals in Canada were recruited during the 2009 H1N1 pandemic. Genomic DNA was extracted from the whole blood samples. The NFkB1 gene was targeted for genotyping using next-generation sequencing technology—Roche 454. Results: A total of 136 single nucleotide polymorphisms (SNPs) were discovered within the NFκB1 gene. Among them, 63 SNPs were significantly enriched in patients admitted in the ICU (*p* < 0.05) compared with the British Caucasian population in the 1000 Genomes study. These enriched SNPs are mainly intron variants, and only two are exon SNPs from the non-transcribing portion of the NFκB1 gene. Conclusions: Genetic variations in the NFκB1 gene could influence clinical outcomes of pandemic H1N1 infections. Our findings showed that sequence variations of the NFκB1 gene might influence patient response to influenza infection.

## 1. Introduction

The spread of Influenza A virus (IAV) H1N1 in 2009 (H1N1pdm09) was one of the most far-reaching examples of a global pandemic and served as an important backdrop to the COVID-19 pandemics. Since 2009, approximately 500,000 people have died from the H1N1 pandemic, with case fatality rate up to 10% among hospitalized patients, which has a higher mortality rate compared to other respiratory virus infections that lead to hospitalization [1,2]. Additionally, a substantial proportion of infected people do not have comorbidities that could explain the severe illness from H1N1pdm09 [1,3].

Significantly elevated cytokine and chemokine levels, the so-called “cytokine storm”, are recognized as the hallmark of acute respiratory viral infections, playing a central role in the severity and lethality of H1N1pdm09 infections [4], with elevated plasma levels of TNF-α, IL1, IL 17, and NFκB1 in a positive feedback manner, leading to inappropriately elevated immune response in the host [5]. This imbalance of pro-inflammatory cytokines can result in lung remodeling, alveolar edema, and severe inflammation of the bronchi. Individuals with cytokine storms exhibit an overwhelming and exaggerated immune response to an infection that would, for most people, lead to much milder reaction. Among host immune responses, the innate immune system plays an important role in the hosts’ resistance to viruses [4,6]. One of the key components within the innate immune system is the NFκB, which plays a central role in the IAV infection. NFkB demonstrated viral supportive and antiviral functions by promoting the transport of the viral genome and by upregulating the host immune system. However, excessive upregulation of NFkB and downstream pro-inflammatory cytokines leads to a severe disease response and high morbidity. In general, IAV exploits the upregulation of NFkB through the consequential upregulation of various TLRs on cellular surfaces, allowing further IAV cellular invasion [7,8,9]. Cellular invasion and insults through TLRs by IAV activate the IKKB pathway, which ultimately activates the NFκB cascade, upregulating various interferons and TNFα, which subsequently activates the immune system in a non-severe disease response [8,9]. The IAV also, however, attempts to control the expression of the NFkB cascade; specifically, the IAV’s NS1 protein selectively inhibits the activation of NFκB1 and the production of NFκB1-induced antiviral genes [10,11]. On the other hand, the NFκB signaling pathway is responsible for the transcription of pro-inflammatory cytokines, where unbalanced expression can lead to lung damage and a severe disease response [12]. It has been shown that inhibition of NFκB impaired IAV replication and cytokine expression such as IL-8, MCP-1, IL-6, RNATES, and IFN-α/β [7,13,14]. Thus, NFκB could be a potential therapeutic target for IAV infection, as it has much higher potential compared to single target approaches to simultaneously inhibit cascades of pro-inflammatory cytokines and chemokines.

Accumulated evidence shows that host genetic factors play important roles in determining the outcomes of H1N1pdm09 infection [15,16,17]. Genetic variations such as single nucleotide polymorphisms (SNPs) in some host genes can contribute to higher levels of transcription, production, and activity of inflammatory and anti-inflammatory cytokines. It has been shown that specific SNPs in pro-inflammatory cytokines are associated with a wide range of infections [18]. A number of SNPs of innate immunity-related genes, including TNFα, IL-17, and IL-2, have been linked to the severity of the influenza illnesses [18,19]. For instance, a commonly occurring variant of IL-1 receptor-associated kinase—a critical regulator of the innate immune system—was associated with sepsis-induced acute lung injury, more severe organ dysfunction, higher mortality, and higher activation of NFκB. Polymorphisms in the TLR3 receptor gene activated the NFκB transcription factor and increased influenza virus replication [12]. Evidently, NFκB is a critical mediator in the innate immune response [10,20,21]. However, there have been rare studies on the genetic variations of the NFκB1 gene and the importance of these genetic variants in contributing to the severity of the influenza disease. As a key innate-immunity-related gene, knowledge of how polymorphisms within the NFκB1 gene affect outcomes of H1N1pdm09 infection could provide insight in detailing how exactly immune response dysregulations contribute to the disease process and contribute to the development of effective therapeutics to prevent and treat the disease.

In this study, we analyzed the NFκB1 gene of 36 Caucasian patients admitted in the intensive care unit (ICU) in several hospitals in Canada and evaluated the associations of the NFκB1 gene SNPs with the risk of severe response to H1N1pdm09 infection. We identified multiple SNPs in the genomic regions of NFκB1 that are enriched in the ICU patients. Our study showed that genetic polymorphism of the NFκB1 gene may influence how patients responded to H1N1pdm09 infection.

## 2. Materials and Methods

### 2.1. The Studied Subjects

A case control study was performed to investigate the relationship and association between NFκB1 gene polymorphism and the severe H1N1pdm09 infection. The cases comprised of 36 Caucasian patients who presented with severe symptoms of acute respiratory tract infection and were recruited from the ICU across Canada during the 2009 H1N1 pandemic. There were no exclusion criteria. All patients were influenza A H1N1-confirmed by RT-PCR testing. The clinical samples, including whole bloods and data, were obtained from the ICUs. The study of ICU patients was approved using a full consent process through the Research Ethics Board (HREB, HS11422). Only anonymous data without patient identifying information were analyzed in this study. Controls comprised 91 British Caucasian individuals from the 1000 Genome Project (build grch37 phase 3) [22].

### 2.2. PCR Amplification and Sequencing of NFκB1 Gene

Genomic DNA was isolated from the collected whole blood samples that were stored in PAXgene Blood RNA tubes (PreAnalytix) using a QIAmp DNA Mini Kit and the EZ1 BioRobot (QIAgen Inc., Mississauga, ON, Canada). The genomic regions of NFκB1 were amplified from the genomic DNAs by overlapping PCRs with the 14 gene-specific pair primers (Supplementary Table S1). The amplified PCR products were confirmed by agarose gel electrophoresis and purified, quantified, and tagged with sequence tags. Sequencing was performed on Genome sequencer FLX from 454 Life Sciences (Roche 454) at Genomic Core, National Microbiology Laboratory (NML). All the sequencing data was submitted to the NCBI BioProject database (Bioproject ID: PRJNA752377).

### 2.3. Sequence Analysis

All raw sequencing reads satisfying the GS FLX default quality screening criteria were decoded by patient-specific multiple identifiers (MIDs) using GS Amplicon Variant Analyzer (454 Life Sciences, Branford, CT, USA). The decoded raw sequencing reads for each patient were then subjected to the second-round quality control (QA) using in-house pipeline. Quality screening with the raw reads was considered valid only if (1). No primer sequences were presented in reads; (2). QA score was more than 20 at each nucleotide or average of each read, and (3). Coverage of each nucleotide/or read was over 30.

The sequencing reads passing QA of each patient were mapped to the reference gene NFκB1 [22], using ‘Bowtie2’ implemented in the Galaxy platform at default settings (Galaxy Version 2.4.2) [23]. SNPs were detected by calling variants using a Bayesian genetic variant detector—‘FreeBayes’ (Galaxy Version 1.3.1) [24]. The identified SNPs were further validated manually in the NFκB1 gene sequence assembly for each patient using ‘Tablet’ [25].

The validated SNPs were annotated with dbSNP information [26]. The frequencies of alleles, including minor allele frequency (MAF) and genotypes, were calculated according to the annotated SNPs of the 36 cases. The annotated SNPs were used to extract the matched identical SNPs and genotypes. The frequencies of alleles and genotypes of the control subjects were extracted from dbSNP or calculated by direct counting based on the extracted SNPs information from the 1000 Genome Project, respectively.

### 2.4. Statistical Analysis

Statistical differences between allele and genotype frequencies were analyzed using chi-square and Fisher exact tests implemented in SPSS for Windows V.13 (Chicago, IL, USA). The false discovery rate was controlled at 0.05 to adjust *p* values for multiple comparisons using the Benjamin-Hochberg (BH) method.

## 3. Results

### 3.1. Population Description

The patient cohort for this study consisted of 36 Caucasian patients who were all hospitalized in the ICU and confirmed to be H1N1 positive. In the patients, 19 (52.78%) were male and 17 (47.22%) were female. The mean ± SD age of the patients was 50.76 ± 14.13 years and the majority of them (86%) were below 65 years old. Two patients (2.57%) were pregnant during the H1N1pdm09 pandemic in our cohort. Almost all the patients (35 out of 36) developed acute respiratory failure and required invasive ventilation. The patients’ mean Apache II score was 22.12 and the estimated death rate was 41.11%. The most prevalent comorbidities were hypertension (47.22%) and diabetes mellitus (25%), followed by cardiovascular diseases (11.11%). Among them, diabetes mellitus was more sensitive to detect mortality (OR = 1.2; 95% CI: 0.79–2.43). Characteristics of the patients were summarized in Table 1.

### 3.2. The Polymorphism of the NFκB1 Gene in the ICU Patients

Of the estimated 117,000 bps of the NFκB1 gene (Chromosome 4: 103421886–103539059), 57,000 bps have been successfully amplified and sequenced, while an estimated 60,000 bps were missing (Supplementary Table S2).

A total of 136 single-nucleotide polymorphisms of NFkB1 gene were identified. Out of the 136 identified mutations, which included insertion, deletion, and polymorphism mutations, 112 matched those annotated in the dbSNP database, and 24 mutations were not found in phase 3 grch37 [26]. Over one-third (38 out of 88) of these annotated SNPs have been reported to be associated with a wide range of diseases, including cancers, heart diseases, hepatitis C, lung diseases, and Crohn’s disease, etc. (Table 2).

**Figure 1:**
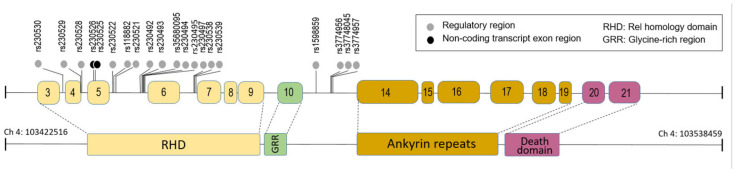
SNP spatial distribution.

The alleles and genotype frequencies for these SNPs in NFκB1 were shown in Table 2 and Table 3. Spatial analysis of the NFκB1 gene along chromosome 4 (Figure 1) highlights the regulatory region and non-transcript exon SNPs. By mapping the non-intron variant SNPs, we see two non-transcript exon variants are very close to each other (50 base pairs). It may be significant that multiple regulatory region variants/SNPs were found before exon 6 on the gene map.

Schematic representation of the spatial locations of regulatory region SNPs and non-transcript exon SNPs. Top image represents the full NFκB1 gene map with locations of regulatory region SNPs and non-transcript exon SNPs indicated. The bottom image represents the protein-coding map, consisting of RHD, GRR, Ankyrin Repeats, and the Death Domain.

Associations among NFκB2 polymorphism genotypes with severe AH1N1pdm09 infection. The *p* value was calculated from Chi-square tests and then adjusted using the Benjamini-Hochberg (BH) method, accounting for 112 SNPs compared. The SNPs with adjusted *p*-value < 0.05 are shown. Ref: NFκB1 reference sequence from 1000GP; Alt: SNPs found in patient as compared to reference; RAF: Heterozygous reference/alt genotype frequency; AAF: Homozygous alt/alt genotype frequency.

### 3.3. Association of SNPs Genotypes with Severe Response to H1N1 Infection

For the 112 SNPs, we compared genotype frequencies of the cases (the 36 Canadian Caucasian ICU patients) with those of the controls (91 controls of British origin in the 1000 Genomes Project). The genotype distributions of these 63 SNPs were found to be significantly different (adjusted *p*-value < 0.05) (Table 3). The genotype distributions for these 63 SNPs demonstrated a homozygous reference/reference (RR) genotype dominance in the cases compared to the controls. For example, in the cases, NFκB1 rs1599961 had RR (81%), heterozygous reference/alternate genotype (RA) (3%), homozygous alternate/alternate genotype (AA) (17%) compared to controls RR (30%), RA (49%), AA (21%). A similar pattern of differences was reported by Chan et al. [15]. Moreover, the allele frequencies were also significantly different for most of the SNPs reported as well, where the major allele frequency (DAF) is consistently higher in the ICU cases in comparison to the controls (Table 2). An example can be seen in the rs1585215 SNP, where the DAF in the ICU cases is 72% in comparison with 62% in the controls.

Furthermore, when clinical and phenotypical characteristic information was matched for the 63 SNPs of the NFκB1 gene via dbSNP, three notable characteristics were found. Most of the SNPs were intron variants, a small proportion of SNPs was located in the regulatory region, and two SNPs were the untranslated exon variants. Each of the 63 SNPs has been reported to be associated with a variety of disease processes across many different organ and physiological systems, as seen in Table 2. Several SNPs were particularly interesting, such as rs170731, rs230528, rs3774956, rs230526, and rs4698858, which are involved in inflammatory reactions, lung disease, and cytokine regulation, respectively [28,29,30].

## 4. Discussion

NFκB is a protein complex that regulates the transcription of a multitude of genes associated with inflammatory cytokines and the downstream interferon-stimulated genes (ISGs) [27]. NFkB1 was intimately involved in the inflammatory cascade and response in respiratory illnesses such as SARS-CoV-2 [9]. Epithelial-immune cell interactions and elevated cytokine and chemokine levels, i.e., cytokine storm, played a central role in the severity and lethality in these respiratory illnesses [12,20,31]. As NFκB1 is a nexus point for multiple redundant inflammatory mediators to start the innate immune response, and genetic variations such as SNPs can contribute to higher levels of transcription, production, and activity of inflammatory as well as anti-inflammatory cytokines, it is hypothesized that individual polymorphic NFκB1 variations could be responsible for the cytokine upregulation, leading to the severe disease response that were seen during the influenza H1N1 pandemic [31,32].

The findings of this study support our hypothesis that NFκB1 gene variation may be a risk factor for developing severe response to H1N1 infection. In this study, we identified 136 SNPs across a variety of locations throughout the NFκB1 gene; 112 were annotated in the dbSNP. Of these 112 SNPs, the major allele frequencies of 63 SNPs were significantly higher in the Caucasian ICU patients compared to the controls (91 British Caucasian subjects without previous health conditions). These 63 SNPs potentially play a role in the severe response to H1N1 infection, since the ICU patients were not in an age group with a high risk of death, nor did they have comorbidities associated with a known increased risk of death from influenza infection (Table 3).

The identified SNPs have been associated with many disease conditions, such as Hodgkin’s lymphoma, colon cancer, ovarian cancer, and many more (Table 2) [33]. Many are involved in various inflammatory reactions that could be a factor in the severe response to the H1N1 infection. Specifically, rs170731, rs230528, and rs4698858 were associated with inflammatory reactions, lung disease, and cytokine regulation, respectively, all of which are crucial in the response to the H1N1 virus [28,29,30]. In addition, SNPs such as rs35680095, rs230528, and rs4698858 were associated with atopic asthma, lung disease, and cytokine regulation. These disease etiologies are all involved in respiratory function and health [28,29,30].

Interestingly, the SNPs identified in the ICU patients were mainly intron variants, and only two are exon SNPs in the non-transcript of the NFkB1 gene, suggesting that most of the associated SNPs with severe response to H1N1 infection may not change the structure of the NFκB1 gene. However, these SNPs may influence NFkB1 expression. For example, the two non-transcript exons SNPs [34,35] were only 50 base pairs apart and might be involved in the expression of exon 5 (Figure 1) [36]. The SNPs such as rs230530, rs230529, rs230528, rs118882, rs230521, rs230493, rs35680095, rs230494, rs230539, rs3774956, and rs4648045 are located in regulatory regions, and a cluster of them are close to the start of exon 6 (see Figure 1); these regulatory region variants had been reported to directly influence the expression of exon 6 and affect the characteristics and function of NFkB1 [37]. These identified SNPs had been reported to be associated with many diseases related with inflammation and immune responses [12,20,38]. Thus, it is possible that the identified SNPs within the NFκB1 gene might influence its expression and cause dysregulation of inflammatory cytokines in H1N1 infection. Future studies of the influence of these SNPs in NFkB1 expression are necessary to validate our findings.

Approximately half of the identified regulatory or exonic region SNPs are very common with higher frequencies in the North American population compared to other populations, including Europeans. For example, the DAF of the rs230530 regulatory SNP (A/G) is much higher in the ICU patients (82%) than the control population (57%) (Table 2). The same is true for rs230529, rs230525, rs35680095, and rs230494. These genetic variants may influence the expression of the NFκB1 gene and their response to H1N1 infection in these Caucasian ICU patients. More population-based studies with much larger sample size are necessary to validate the association between these NFkB1 polymorphisms and the severe response to H1N1 infection.

The strength of our study comes from using high throughput NGS technology, Roche 454, to sequence the full NFκB1 gene. NGS systems have an incredibly high resolution that allows precise regional amplification and deep coverage with low error probabilities in identifying SNPs [39]. Importantly, the mentioned benefits also allow the discovery of genetic variants that do not exist in the established database. We have identified 24 novel SNPs, which may be researched further to understand the polymorphism of NFκB1 in the severe H1N1 response and other diseases. Moreover, the addition of the novel SNPs in the SNP compendium will make it possible to create meta-analyses such as studying gene linkages and multiple SNP interactions within the gene itself [35,40,41]. The limitations of this study include a small sample size, which may reduce the statistical power for the identification of SNPs associated with the severe H1N1 infection. Calculating and presenting the false discovery rate according to the *p*-value and the multiple comparison method using BH would increase the accuracy of the identification of SNPs associated with outcomes of H1N1 infection. Secondly, ages of controls are missing, which is a confounding factor affecting outcomes of H1N1 infection. Lastly, there is the uncertainty of the infection outcomes among the controls that may affect our results, although ICU rates are less than 1% of H1N1-infected infection. More studies using large sample size and moderately ill patients as controls are required to validate the results.

In conclusion, our results suggest that NFκB1 polymorphisms may influence the risk of severe H1N1 infection. The impacts of NFκB1 genetic polymorphism on the risk of severe disease should be further elucidated. Our findings suggest that the NFkB1 gene expression may be a potential target for the treatment of critical stage H1N1 patients, since “NFkB controls the transcription of a large number of genes associated with inflammatory cytokines and the downstream ISGs” [27]. Targeting the NFkB1 expression may prevent dysregulation of multiple cytokines and/or chemokines in patients with severe response to future influenza infections.

## Figures and Tables

**Table 1:** Characteristics of the studied patients with confirmed H1N1_pdm09_.

Variables	H1N_1pdm09_ (n = 36)
Ethnicity (%)	Caucasian (100%)
Gender	
Male	19 (52.78%)
Female	17 (47.22%)
Age (years)	
<25	2 (5.67%)
25–49	12 (33.33%)
50–64	17 (47.22%)
>65	5 (13.89%)
Average	50.76 + 14.13
Pregnancy	2 (5.67%)
Comorbidity	
Obesity	5 (13.89%)
Diabetes Mellitus	9 (25.00%)
Hypertension	17 (47.22%)
Coronary heart disease	1 (2.78%)
Congestive heart failure	3 (8.33%)
Chronic obstructive	5 (13.89%)
pulmonary disease	
Asthma	7 (19.44%)
Immunosuppression	1 (2.78%)
Invasive Ventilation (%)	
Apache II Score	22.12 ± 6.79
Death Rate	0.41 ± 0.19

Pdm09: pandemic 2009; Apache II: the acute physiologic assessment and chronic health evaluation II. Ave: average.

**Table 2:** Allele frequencies and association with severe AH1N1pdm2009 for SNPs previously reported to be associated with diseases.

SNP ID	Chromosome Location	Minor Allele	MAF ^a^		*p* Value	Phenotype ^d^
			ICU ^b^	GBR ^c^		
rs1599961	103443569 (intronic)	G	0.18	0.46	1.53 × 10^−7^	Cardiovascular, hypertension,
rs1585215	103444474 (intronic)	C	0.28	0.38	1.53 × 10^−2^	Hodgkin lymphoma
rs1585213	103444698 (intronic)	T	0.38	0.46	9.78 × 10^−3^	measles vaccine effectiveness, various diseases
rs1598856	103446115 (intronic)	A	0.50	0.57	4.82 × 10^−2^	Cholangitis, ovarian cancer, gastric cancer
rs230535	103448582 (intronic)	A	0.51	0.62	6.11 × 10^−3^	Bipolar disease
rs170731	103448903 (intronic)	T	0.39	0.62	1.30 × 10^−6^	lymphedema, inflammatory reactions
rs230534	103449041 (intronic)	T	0.32	0.62	3.36 × 10^−8^	Maculopathy, biliary and crohn’s disease
rs230533	103450083 (intronic)	A	0.44	0.62	8.10 × 10^−5^	crohn’s disease
rs230532	103450167 (intronic)	T	0.54	0.62	3.83 × 10^−2^	Hepatitis C, PBC, breast cancer inflammation
rs230531	103450377 (intronic)	G	0.35	0.62	2.24 × 10^−9^	PBC, ovarian cancer, gastric cancer
rs230530	103453980 (regulatory)	G	0.18	0.43	2.41 × 10^−3^	Hepatitis C, gastric cancer, allergic rhinitis
rs230529	103457418 (regulatory)	C	0.19	0.55	2.09 × 10^−7^	drug addictions, schizophrenia, psychosis
rs230528	103457585 (regulatory)	G	0.19	0.55	1.60 × 10^−8^	lung disease, coronary artery disease
rs230526	103458825 (exonic)	G	0.25	0.55	7.88 × 10^−5^	metabolic variation, cytokine variation
rs230525	103458877 (exonic)	G	0.38	0.62	3.08 × 10^−5^	liver cancer
rs4648004	103461107 (intronic)	G	0.11	0.32	6.08 × 10^−3^	cerebrovascular, cardiovascular
rs230523	103462038 (intronic)	C	0.11	0.62	0.00 × 10^0^	various diseases
rs118882	103463007 (regulatory)	T	0.39	0.62	1.23 × 10^−6^	allergic rhinitis
rs230521	103463328 (regulatory)	C	0.26	0.55	6.59 × 10^−5^	breast cancer, hepatitis c, colorectal cancer,
rs230520	103465612 (intronic)	G	0.44	0.62	6.27 × 10^−4^	PBC
rs230519	103466749(intronic)	T	0.44	0.62	6.10 × 10^−4^	PBC, Tuberculosis
rs93059	103468518 (intronic)	A	0.11	0.42	1.51 × 10^−5^	various diseases
rs230493	103486216 (regulatory)	A	0.42	0.62	2.89 × 10^−4^	thyroid cancer, PBC, antipsychotic response
rs35680095	103486698 (regulatory)	A	0.03	0.12	3.66 × 10^−2^	atopic asthma
rs230494	103486969 (regulatory)	A	0.29	0.55	7.03 × 10^−4^	various diseases
rs230496	103488491 (intronic)	G	0.31	0.55	3.16 × 10^−3^	liver cancer
rs230498	103489603 (intronic)	A	0.44	0.63	2.89 × 10^−3^	ovarian cancer, gastric cancer
rs230500	103491724 (intronic)	A	0.40	0.63	8.34 × 10^−5^	cytokine regulation
rs230539	103495532 (regulatory)	G	0.36	0.6	3.24 × 10^−8^	ovarian cancer, gastric cancer
rs1598858	103506095 (intronic)	G	0.18	0.4	1.82 × 10^−4^	kidney inflammation, cytokine regulation,
rs3774956	103508526 (regulatory)	T	0.31	0.48	3.77 × 10^−3^	lymphedema, cytokine regulation,
rs4648045	103508703 (regulatory)	C	0.29	0.41	3.80 × 10^−3^	various diseases
rs3774959	103511114 (intronic)	A	0.19	0.41	4.48 × 10^−5^	Asthma, glioma, cytokine regulation,
rs4698858	103513073 (intronic)	-	0.36	0.48	3.62 × 10^−2^	cytokine regulation
rs11722146	103524629 (intronic)	-	0.19	0.35	3.24 × 10^−3^	colon and rectal cancer, colorectal cancer,
rs12509403	103525350 (intronic)	T	0.18	0.35	7.08 × 10^−4^	kidney inflammation, allergic rhinitis
rs12509517	103525508 (intronic)	C	0.19	0.35	3.18 × 10^−3^	colorectal cancer, gastric cancer
rs3774968	103531112 (intronic)	A	0.49	0.6	2.84 × 10^−3^	plasma cell leukemia, vte, ovarian cancer,

a: minor allele; b: minor allele frequencies. c: DAF—Major allele frequencies c: GBR—British Caucasian population from 1000 Genomes. d: Data obtained from Ensemble Variant Effect Predictor (VEP) grch37 release 104 [27]. The allele frequencies reported in the 36 Canadian Caucasian cases were compared to the 91 controls from the 1000 genomes for the British Caucasian population. The adjusted *p* value was calculated from Fisher Exact Test, accounting for the 112 multiple comparisons across the SNPs found in the dbSNP using the Benjamin-Hochberg method.

**Table 3:** Associations of NFKB1 genotype frequencies with risk of severe AH1N1_pdm09_ infection.

SNP ID	Alleles (Ref/Alt)	ICU		GBR		Adjusted *p* Value	SNP ID	Alleles (Ref/Alt)	ICU		GBR		Adjusted *p* Value
		RAF	AAF	RAF	AAF				RAF	AAF	RAF	AAF	
rs1599961	G/A	0.03	0.2	0.5	0.2	1.53 × 10^−7^	rs230497	T/A	0.06	0.2	0.5	0.4	0.00 × 10^0^
rs1585215	T/C	0.22	0.2	0.5	0.1	1.53 × 10^−2^	rs230498	A/G	0.28	0.3	0.5	0.4	2.89 × 10^−3^
rs1585214	T/C	0.08	0.3	0.5	0.3	2.29 × 10^−6^	rs230499	T/G	0.06	0.3	0.5	0.4	5.60 × 10^−9^
rs1585213	C/T	0.19	0.3	0.5	0.2	9.78 × 10^−3^	rs230500	A/G	0.19	0.3	0.5	0.4	8.34 × 10^−5^
rs1598856	A/G	0.28	0.4	0.5	0.3	4.82 × 10^−2^	rs230502	A/G	0.25	0.1	0.5	0.2	9.77 × 10^−4^
rs230535	A/C	0.25	0.4	0.5	0.4	6.11 × 10^−3^	rs230503	G/A	0.25	0.3	0.5	0.4	1.96 × 10^−3^
rs170731	T/A	0.11	0.3	0.5	0.4	1.30 × 10^−6^	rs230538	C/T	0.17	0.2	0.5	0.3	4.38 × 10^−5^
rs230534	T/C	0.08	0.3	0.5	0.4	3.36 × 10^−8^	rs230539	G/A	0.06	0.3	0.5	0.3	3.24 × 10^−8^
rs230533	A/G	0.17	0.4	0.5	0.4	8.10 × 10^−5^	rs1598858	A/G	0.14	0.1	0.5	0.1	1.82 × 10^−4^
rs230532	T/A	0.31	0.4	0.5	0.4	3.83 × 10^−2^	rs1598859	T/C	0.28	0.1	0.5	0.1	3.44 × 10^−2^
rs230531	G/A	0.03	0.3	0.5	0.4	2.20 × 10^−9^	rs3774956	C/T	0.22	0.2	0.5	0.2	3.77 × 10^−3^
rs230530	A/G	0.31	0	0.5	0.2	2.41 × 10^−3^	rs4648045	T/C	0.19	0.2	0.5	0.1	3.80 × 10^−3^
rs230529	T/C	0.11	0.1	0.5	0.3	2.09 × 10^−7^	rs3774957	A/T	0.19	0.2	0.5	0.1	2.87 × 10^−3^
rs230528	G/T	0.06	0.2	0.5	0.3	1.60 × 10^−8^	rs1287	G/A	0.03	0.2	0.5	0.2	5.88 × 10^−8^
rs230527	A/G	0.22	0.2	0.5	0.4	6.11 × 10^−6^	rs7692606	T/C	0.19	0.2	0.5	0.1	2.81 × 10^−3^
rs230526	A/G	0.22	0.1	0.5	0.3	7.88 × 10^−5^	rs230546	G/C	0	0.8	0	1	1.00 × 10^−3^
rs230525	G/A	0.19	0.3	0.5	0.4	3.08 × 10^−5^	rs647417	A/T	0	0.8	0	1	8.16 × 10^−5^
rs79651301	G/A	0.17	0	0.3	0.1	3.11 × 10^−2^	rs647424	G/T	0	0.6	0	1	3.50 × 10^−8^
rs4648004	A/G	0.22	0	0.4	0.1	6.08 × 10^−3^	rs1020759	C/T	0	0	0.5	0.2	0.00 × 10^0^
rs230523	C/T	0	0.1	0.5	0.4	0.00 × 10^0^	rs3774959	G/A	0.11	0.1	0.5	0.1	4.48 × 10^−5^
rs230522	A/G	0.11	0.3	0.5	0.4	5.24 × 10^−7^	rs3774960	C/T	0.25	0.3	0.5	0.2	2.31 × 10^−2^
rs118882	T/C	0.11	0.3	0.5	0.4	1.23 × 10^−6^	rs4698858	C/G	0.28	0.2	0.5	0.2	3.62 × 10^−2^
rs230521	C/G	0.19	0.2	0.5	0.3	6.59 × 10^−5^	rs7684888	G/A	0.03	0.1	0.4	0.1	2.34 × 10^−5^
rs230520	G/A	0.22	0.3	0.5	0.4	6.27 × 10^−4^	rs11722146	G/A	0.11	0.1	0.4	0.1	3.24 × 10^−3^
rs230519	T/C	0.22	0.3	0.5	0.4	6.10 × 10^−4^	rs12509403	C/T	0.08	0.1	0.4	0.1	7.08 × 10^−4^
rs93059	G/A	0.17	0	0.5	0.2	1.51 × 10^−5^	rs12509517	G/C	0.11	0.1	0.4	0.1	3.18 × 10^−3^
rs230492	A/G	0.22	0.3	0.5	0.4	6.75 × 10^−4^	rs3774967	T/C	0.14	0.1	0.4	0.1	1.04 × 10^−2^
rs230493	A/T	0.22	0.3	0.5	0.4	2.89 × 10^−4^	rs4699031	A/G	0.14	0.1	0.4	0.1	1.03 × 10^−2^
rs35680095	G/A	0.06	0	0.2	0	3.66 × 10^−2^	rs4235406	T/C	0.11	0.2	0.4	0.1	3.92 × 10^−3^
rs230494	G/A	0.25	0.2	0.5	0.3	7.03 × 10^−4^	rs9790601	A/G	0.06	0.1	0.4	0.1	9.49 × 10^−5^
rs230495	A/G	0.31	0.2	0.5	0.3	5.60 × 10^−3^	rs3774968	A/G	0.19	0.4	0.5	0.4	2.84 × 10^−3^
rs230496	G/A	0.39	0.1	0.5	0.3	3.16 × 10^−3^							

## Data Availability

All the sequencing data for this study can be found in the NCBI BioProject database (Bioproject ID: PRJNA752377 (Available online: http://www.ncbi.nih.gov/bioproject/).

## Supplementary Materials

The following supporting information can be downloaded at: https://www.mdpi.com/article/10.3390/microorganisms10101886/s1. Table S1: NFκB1 primers used for the amplification of sequences via PCR; Table S2: NFκB1 successful and failed regions of amplifications by PCR sequencing.
