# Supplementary material for: NFκB1 Polymorphisms Are Associated with Severe Influenza A (H1N1) Virus Infection in a Canadian Population
Source: Microorganisms. 2022 Sep 21;10(10):1886. doi: 10.3390/microorganisms10101886 (PMC9606957; doi:10.3390/microorganisms10101886)
Supplement: Supplementary file 1 [file microorganisms-10-01886-s001.zip › microorganisms-1855842-supplementary.pdf]

Supplementary Table S1. NFκB1 primers used for the amplification of sequences via PCR

Name	Location Ch4	Sequence	# nt	Amplicon	Tm	PCR Ta
NFKB1_F1	103422079	AGGGGCTATGGACCGCATGACTCTATC	27	5531	70.7	**64.2**
NFKB1_R1	103427581	GCTTGTTCCTGAGGTTTGCCATCTAGTAAC	30		69.2	
NFKB1_F2	103426222	CCTTTGGTCTCTGCTGTATTAGGGGTTG	28	9670	68.7	**61.3**
NFKB1_R2	103435864	GGCAGAACTAATGGAAAAAATGACAAATC	29		66.3	
NFKB1_F3	103434399	TGGAAATGGTAGGCAGCAATATCTCAG	27	9082	67.1	**62.1**
NFKB1_R3	103443453	GCTGAGGTAACAGTAACGCTATGGTGATG	29		67.8	
NFKB1_F4	103442464	CTCACATTCTTTATCAGTTTATTAGCCAGCAG	32	9357	67.1	**62.1**
NFKB1_R4	103451795	ATGCCTCCTTCAGCGATTACCAAACTC	27		69.2	
NFKB1_F5	103450287	CTTTTGTCATATAAGGTAGCACTCACAGTTTC	32	10615	65.4	**60.0**
NFKB1_R5	103460877	TCCATTCTCTGAGTCTGTTTGGGTTC	26		65.0	
NFKB1_F6	103459060	GGACTACCTGGTGCCTCTAGTGAAAAGAAC	30	9956	68.5	**61.5**
NFKB1_R6	103468987	ATCTCAGTCTCCTATCTTAACCCCAGACAG	30		66.5	
NFKB1_F7	103468081	CCCTGAATAGAGTAGGAAACCCCAAGTC	28	9972	67.3	**61.4**
NFKB1_R7	103478026	CCCTTCTGGATAATGATGATGACAACTG	28		66.4	
NFKB1_F8	103477170	GAATGAGAATGGAAGAAAGGGACAAACTG	29	9366	68.0	**63.0**
NFKB1_R8	103486149	CGTCAACAAAGGACAGACTCCCCTACAG	28		69.5	
NFKB1_F9	103485656	CAGATGATGCGATGCTGACTTGGACTC	27	10080	70.1	**64.7**
NFKB1_R9	103495709	GGCAATCTCTGAGGTTGAGGAGACACAG	28		69.7	
NFKB1_F10	103494673	CATAGTTTCCTTCATGTTGCTTGTGCTTG	29	10597	68.6	**62.2**
NFKB1_R10	103505241	GTATTCTCTTTGACATTCTTGTTCCCCATC	30		67.2	
NFKB1_F11	103504248	TGTCTCAACCTACCAAATATCTGGGACTAC	30	10123	65.9	**60.9**
NFKB1_R11	103514342	CAGTACACACCCAAATCCACTCTACAACAG	30		67.6	
NFKB1_F12	103513617	CAACATTGAGGCAAGACCTTCCACCAG	27	9895	70.9	**65.2**
NFKB1_R12	103523484	GGTTTCACCATGTTAGCCAGGATGATCTC	29		70.2	
NFKB1_F13	103522537	GAAGTCTACTGAAACATACCTTGACAACATC	31	9410	64.1	**59.1**
NFKB1_R13	103531924	GGAATAAAGCACCAGGAAGACCATC	25		64.5	
NFKB1_F14	103531644	GCTTTGCCTTTGGGAATCTGACCTTTG	27	7190	71.2	**65.1**
NFKB1_R14	103538809	CTCCACATCCTCTCCAGCACCTGTTG	26		70.1	

NFKB1: nuclear factor Kapa B subunit 1; F: forward; R: reverse; nt: nucleotide; Tm: the primer melting temperature; Ta: the primer annealing temperature.

Supplementary Table S2. NFκB1 successful and failed regions of amplifications by PCR sequencing

Missing Locations	Amplified Locations
• 103421886-103436285	• 103436285-103436585
• 103436585-103442485	• 103442485-103468985
• 103468985-103485685	• 103485685-103495785
• 103495785-103504285	• 103504285-103514385
• 103514385-103522585	• 103522585-103531985
• 103531985-103539059	
